# Population‐Level Awareness of Water Safety Messages: An Analysis of a Representative Sample From New South Wales, Australia

**DOI:** 10.1002/hpja.70261

**Published:** 2026-10-18

**Authors:** Amy E. Peden, Jasmin C. Lawes, Rachel J. Irvine

**Affiliations:** ^1^ School of Population Health UNSW Sydney Kensington New South Wales Australia; ^2^ UNSW Beach Safety Research Group UNSW Sydney Kensington New South Wales Australia; ^3^ School of Biological, Earth and Environmental Sciences UNSW Sydney Kensington New South Wales Australia; ^4^ Surf Life Saving Australia Bondi Beach New South Wales Australia

**Keywords:** communication, drowning prevention, education, health promotion, social marketing

## Abstract

**Issue Addressed:**

The World Health Organisation recommends strengthening public awareness of drowning through strategic communications as a key drowning prevention strategy. This study aimed, for the first time, to assess population‐level awareness of water safety messages in New South Wales, Australia.

**Methods:**

Via an age‐sex representative sample survey, community beliefs around the number of water safety messages and awareness of messages (defined as having seen or heard) were examined. Message awareness variance between different demographics (gender, age group, country of birth, rurality and socio‐economic classification of residence) and water use frequency groups was assessed using chi square tests.

**Results:**

The sample comprised 1495 people (52% female; 33% 45–64 years; 84% Australian‐born and 78% metropolitan residents). Seventy‐six percent did not believe the number of water safety messages promoted to the community was confusing. ‘Swim between the flags’ had the highest awareness among the sample (68%), followed by ‘No flags, no swim’ (53%). Despite no longer being actively promoted, 36% were aware of the message ‘To escape a rip, swim parallel to the beach’. ‘Respect the River’ had significantly higher awareness among males, Australian‐born and regional residents, at‐risk cohorts for river drowning.

**Conclusions:**

Message longevity supports increased awareness, though awareness varies by message and demographic groups. Funding support to increase awareness should reflect drowning burden. So what? Funding, time, and specific calls to action will support moving from awareness to behaviour change.

## 1 Introduction

Drowning is a leading cause of injury‐related mortality and morbidity globally, and in Australia [1, 2]. Long‐term data analysis [3] shows an average of 284 drowning deaths annually over the last 10 years [2]. The 313 drowning deaths recorded nationally in the 2025/26 financial year represent a 10% increase on the 10 year average [2].

As a preventable cause of health‐related harm, there are a range of interventions and strategies that are recommended to reduce drowning risk at the national or community level [4]. Among the interventions and strategies recommended by the World Health Organisation (WHO) is a focus on strengthening public awareness of drowning through strategic communications, such as drowning prevention mass media campaigns and awareness campaigns on alcohol use near water [5]. Efforts that focus on enhancing public awareness and behaviour change campaigns are recommended to encourage drowning prevention measures to be more widely accepted and successfully adopted among the community [4]. Alongside development and implementation, the WHO encourages evaluation of any campaign or message to determine efficacy [4].

Australia has a long history of drowning prevention with achievements made in reducing drowning fatality rates among high‐risk cohorts including children under five [6, 7]. Central to this approach has been legislative change and government support of interventions implemented at both the national and sub‐national levels, complemented by numerous public education and awareness raising programs and campaigns such as Keep Watch and Kids Alive Do The Five, among others [3, 8].

Despite WHO recommendations, to date, there has been limited evaluation of drowning prevention messages globally [9]. In Australia, focus is often placed on evaluations conducted on single campaigns or demographic groups. One evaluation of a video aimed at men discouraging alcohol consumption around water found short‐term positive effects for both males' own behaviours and encouraging safer behaviours among their peers [10]. An evaluation of a flood safety video infographic which aimed to discourage people from entering floodwaters in their vehicle found positive immediate post‐campaign impacts on attitudes and subjective norms of both men and women, but only changes in beliefs among women were maintained over time [11].

The evaluation of two television commercials screened in Western Australia, promoting the supervision of children in and around water, found modest awareness (32% of the sample) and high comprehension (82%–83%). Among those who comprehended the message, approximately half of the sample (50%) self‐reported intending to take action regarding child supervision around water [12]. Evaluation of ‘The Think Line’, a behaviour change campaign focusing on encouraging beachgoers to stop, look and plan when it comes to rip currents, found 83% of respondents to a survey who were shown campaign material self‐reported being more likely to swim between the red and yellow flags in the future, and the same proportion reported being more likely to stop and look for rip currents before entering the water [13].

In the transtheoretical model of behaviour change, awareness is an important first step to improving safer behaviours around water and reducing drowning mortality and morbidity [14]. Given the paucity of existing studies and the significant investment in drowning prevention campaigns and messages by government and non‐government organisations, this study examines overall awareness of water safety messages among the Australian population by different demographic and water use groups. Findings can inform future efforts, including identifying populations where awareness of particular messages could be improved given drowning risk, as well as inform future resource allocation for water safety messaging. Further, given an anecdotal narrative exists within the Australian water safety community that there may be too many water safety messages promoted to the public, a secondary aim was to determine community views regarding the number of water safety messages promoted and any potential confusion arising from this.

## 2 Methods

This study is a quantitative analysis of a survey of a representative sample of the New South Wales population conducted in 2025. The survey was initially undertaken as part of a broader evaluation of a state‐wide water safety public awareness campaign—Stop. Look. Stay Alive [15, 16] which explored the feasibility and acceptability, from the perspective of multiple water safety organisations and the public, of standardised messaging across coastal, inland and pool environments.

### 2.1 Survey Design

The survey was designed based on the drowning prevention and water safety expertise of the authorship team, including their awareness (defined as having seen or heard) of water safety messages promoted to the community. The authorship team has a combined 35 years of experience in drowning prevention including in practitioner and academic research roles predominately in high income contexts, albeit with some expertise in low and middle income countries in South East Asia. The full survey contained 25 questions (predominately closed questions, with 5 open‐ended questions) and took approximately 10 min to complete. The survey started with basic demographic and water activity participation questions (type and frequency) as well as knowledge of existing water safety messages. These were followed by specific questions about participant knowledge of, engagement with, and opinions of the ‘Stop. Look. Stay Alive.’ water safety message.

Given a long history of drowning prevention and water safety messages in New South Wales, a select number of messages were chosen to test community awareness. These included long‐standing and more recent coastal‐specific safety messages and a selection of water safety messages aimed at diverse aquatic environments. See Box 1 for a list of the coastal‐specific and non‐coastal‐specific messages included in this study, the duration of the message and the goal.

BOX 1 **Information about water safety messages assessed in this study.**

**Coastal‐Specific Messages**

- Don't Put Your Life on the Line (2014‐ongoing): Encourages rock fishers to ensure they return home safely including encouraging fishers to wear a lifejacket, check conditions before fishing and never turn their back on the ocean.
- Give an F About the Flags (2023–2024): Urges young males in particular to swim only between the red and yellow flags at patrolled locations.
- No Flags, No Swim (Unknown): to discourage swimmers from entering the water at unpatrolled beaches or outside of the patrol area.
- Swim Between The Red and Yellow Flags (1935‐ongoing): to encourage beachgoers to swim at patrolled beaches between the flags to ensure lifesaver or lifeguard supervision.
- The Think Line: Stop, Look, Plan (2016–2023): to encourage unpatrolled beach users to avoid entering a rip and have a plan to ensure their safety.
- To Escape a Rip, Swim Parallel to the Beach (2009–2013): to highlight how to escape a rip to avoid being taken out to sea (no longer promoted)

**Non‐coastal specific messages**

- Float to Survive (2022‐ongoing): encouraging those who get into trouble in the water to stay calm and float while awaiting assistance.
- Keep Watch (2000‐ongoing): to highlight four key child drowning prevention strategies to parents and caregivers (supervision, restricting access, water awareness, and resuscitation).
- Make the Right Call (2020‐ongoing): aimed specifically at men encouraging safer choices around the water. The campaign features mates encouraging each other to avoid alcohol and wear a lifejacket.
- Respect the River (2015‐ongoing): highlights that rivers are a leading drowning location and should not be underestimated given their often calm and still appearance. The campaign draws attention to hazards hidden under the water's surface, such as currents, undertows, and submerged objects.
- Stop. Look. Stay Alive (2024‐ongoing): to encourage beachgoers, boaters, rock fishers, and river and pool users to have a plan before heading out to ensure they are as safe as possible.

### 2.2 Data Collection

Data collection was conducted by the market research company Dynata who hosted and distributed the survey to their consumer panel. Participants were paid a small incentive for completing the survey. The sample was restricted to NSW residents aged 18 and older and was representative of the broader population based on age and gender quotas. Data collection ran from 14 to 22 May 2025 and closed once a quota of 1500 surveys was surpassed, finishing with a total of 1538 surveys.

### 2.3 Data Cleaning and Analysis

Data cleaning comprised the removal of 43 respondents due to incomplete responses to the gender/sex and/or postcode question(s). Water safety message questions asked ‘There are a number of water safety messages that are being promoted across Australia. Do you find the number of messages to be confusing to you?’ with answer options being *yes*, *no*, and *unsure*. If *yes*, respondents were invited to provide an open text answer to the following question ‘Why do you find the number of messages confusing?’ This was followed by a randomised list of messages (see Box 1) and a question that asked ‘Which of the following water safety messages have you seen/heard about before?*’* (select all that apply).

Demographic variables of interest included gender (dichotomised to female/male) and age group (originally framed as 18–19, 5‐year age groups between 20 and 74, with 75+ years, which were condensed into four age groups 18–29, 30–44, 45–64 and 65+ years). Country of birth was dichotomised into Australia and Overseas. Respondents were then asked to enter their residential postcode, which was then used to code rurality of residence using the Australian Statistical Geography Standard (ASGS) 2021 [17] with five classifications dichotomised to metropolitan (major cities) or regional (inner and outer regional and remote and very remote). (Table 1).

**TABLE 1:** Questions, variables and coding used in analysis.

Focus questions and variables	
	Responses
Demographics	
Gender^a^	Male
	Female
	Non‐binary/Third gender
	Prefer not to say
	Prefer to give own answer
Age group^b^	18–19
	20–24
	25–29
	30–34
	35–39
	40–44
	45–49
	50–54
	55–59
	60–64
	65–69
	70–74
	75+
Country of birth	Australia
	Overseas
Rurality of residential postcode	Metropolitan
	Regional/Remote
Water usage Q: ‘In what ways did you interact with the water this summer? (daily, 2–3 times a week, weekly, 2–3 times a month, monthly, did not do)^c^	Swimming at the beach
	Surfing/bodyboarding
	Swimming in a river
	Swimming in a pool
	Boating
	None
Message confusion Q: There are a number of water safety messages that are being promoted across Australia. Do you find the number of messages to be confusing to you?	Yes
	No
	Unsure
If yes, why do you find the number of messages confusing?	Free text
Messages Which of the following water safety messages have you seen/hard about before?^d^	Don't put your life on the line
	Give an F About the Flags
	No Flags, No Swim
	Swim Between the Red and Yellow Flags
	The Think Line: Stop Look Plan
	To Escape a Rip, Swim Parallel to the Beach
	Float to Survive
	Keep Watch
	Make the Right Call
	Respect the River
	Stop. Look. Stay Alive
	Don't know any messages

^a^ Grouped into Male, Female.

^b^ Grouped into 18–29 years, 30–44 years, 45–64 years, 65+ years.

^c^ Grouped into frequently (daily or 2–3 times a week), infrequently (weekly to monthly) or did not use.

^d^ Grouped into coastal specific messages and non‐coastal specific messages.

Water usage variables of interest were responses to the question ‘In what ways did you interact with the water this summer and how frequently?’ with water usage type (*swimming/wading at the beach*; *surfing/bodyboarding at the beach*; *swimming/wading at a river*; *swimming in a pool*; *boating*) dichotomised into *yes* (*daily*, *2–3 times a week*, *weekly*, *2–3 times a month*, *monthly*) or *no* (*did not do*).

Summer water usage frequency was determined based on the respondents' highest frequency response, for example, if they reported being frequent pool swimmers, they were a frequent water user no matter what their responses to other water activity questions. Frequent water users were defined as having completed a water activity daily or 2–3 times a week, infrequent water users completed a water activity weekly to monthly, and those that did not participate in a single water activity were coded as did not do.

Respondents were invited to provide a written response if they agreed that the number of water safety messages was confusing, and these were analysed thematically. Of the 187 open answer responses, 69 were removed as they were either incoherent or unrelated to the posed question, leaving 118 responses. Inductive coding [18] was performed independently by two authors (AP and RI), who then met to determine broader themes. Direct quotes are used, attributed to the respondents' age group, gender, remoteness classification of residence and whether they were born in Australia or overseas.

Data were analysed using Microsoft Excel (v. 16.97) and R‐studio (v 2026.1.1.403). Non‐parametric Chi‐Square tests examined overall awareness of messages, while Pearson Chi‐Square tests of association were conducted to examine awareness of messages by demographic variables. Statistical significance was set at *p* < 0.05. A modified Bonferonni correction [19] was applied where category included more than two variables.

### 2.4 Ethics

Human research ethics approval was granted by UNSW Sydney Human Research Ethics Committee (approval number: iRECS8327). Informed participant consent was gained prior to the administration of the survey.

## 3 Results

### 3.1 Demographic Characteristics

A total of 1495 responses were analysed. Characteristics of the analysed sample are shown in Table 2. Slightly more than half were female (51.6%; *n* = 772), with the largest proportion aged 45–64 years (33.0%; *n* = 496). The vast majority of respondents were born in Australia, with just 16.5% born overseas. Among those born overseas, the most common countries were the United Kingdom (2.8%; *n* = 42), India (2.5%; *n* = 38), New Zealand (1.2%; *n* = 18), and China (1.0%; *n* = 15). Almost three quarters of respondents (73.3%) resided in metropolitan areas. Swimming at the beach and in a pool (75.5% each respectively) were the most popular Summer activities, followed by swimming in a river (43.5%) (Table 2).

**TABLE 2:** Demographics of sample population (*N* = 1495).

	Number	Percentage (%)
Total	1495	100.0
Gender		
Female	772	51.6
Male	723	48.4
Age group		
18–29 years	286	19.0
30–44 years	436	29.0
45–64 years	496	33.0
65+ years	277	18.0
Country of birth		
Australia	1249	83.5
Overseas	246	16.5
Rurality of residence		
Metropolitan	1096	73.3
Regional	399	26.7
Summer water usage type*		
Swimming at the beach	1129	75.5
Surfing/bodyboarding	565	37.8
Swimming in a river	650	43.5
Swimming in a pool	1128	75.5
Boating	559	37.4
None	201	13.4
Summer water usage frequency		
Frequently (daily or 2–3 times a week)	541	36.2
Infrequently (weekly to monthly)	753	50.4
Did not do	201	13.4

*Note:* * = does not total 100% as question was multi‐select.

### 3.2 Message Confusion

When asked if the number of water safety messages was confusing, 75.7% (*n* = 1132) of respondents indicated that it was not, while 12.5% (*n* = 187) indicated that it was and 11.8% (*n* = 176) reported being unsure. Younger respondents (18–29 and 30–44 years; [*X* ^2^ = 43.14; *p* < 0.001]) and respondents residing in metropolitan areas (*X* ^2^ = 6.860; *p* = 0.009) reported significant confusion relating to the number of water safety messages. There were no significant differences in confusion about the number of messages by respondent gender or country of birth.

As described below, two themes emerged from the written responses of those who found the number of messages confusing.

#### 3.2.1 Theme 1: ‘Not sure what to do, which to follow and when to do it’

For many respondents, the number of messages was found to be contributing to confusion, with overlapping messages making it hard to know what was most important: ‘There are too many water‐safety promotions with overlapping and inconsistent messages, making it hard to distinguish key safety tips’ (18–29 years, Female, Metropolitan, Australian Born). Confusion was exacerbated when messages were perceived to be contradictory: ‘There isn't clear indicators of what the messages mean and they sometimes contradict one another’ (18–29 years, Male, Metropolitan, Australian Born). Messages were also found to be too colloquial: ‘too much lingo and lack of detail’ (30–44 years, Male, Regional, Australian Born) or did not provide sufficient detail for people to enact: ‘they are not taught or very self explanatory’ (18–29 years, Female, Metropolitan, Australian Born).

#### 3.2.2 Theme 2: ‘Who or what to trust?’

Many respondents indicated that the number of messages and the different organisational sources made it hard to know which messages should be prioritised or trusted: ‘Sometimes, when the audience sees the information, it is difficult for them to judge its authority’ (18–29 years, Male, Metro, Australian Born). The overlapping roles of existing authorities caused confusion with suggestions for refinement: ‘Too many groups sending messages. There should be one controlling body to promote water awareness’ (45–64 years, Male, Regional, Australian Born).

### 3.3 Message Awareness

Non‐parametric testing indicated significant awareness of two messages ‘Swim Between the Red and Yellow Flags’ (68.0%; *n* = 1016; *X* ^2^ = 192.89; *p* < 0.001) and ‘No Flags, No Swim’ (53.3%; *n* = 797; *X* ^2^ = 6.556; *p* = 0.01) (Table 3). ‘Keep Watch’ was the non‐coastal‐specific message with the highest awareness (27.4%; *n* = 410), followed by ‘Float to Survive’ (21.8%; *n* = 326) and ‘Respect the River’ (19.0%; *n* = 284) (Table 3). Just 4.6% of respondents reported not being aware of any of the messages.

**TABLE 3:** Awareness of coastal‐specific water safety messages by demographic and water usage.

	Swim between the flags			The Think Line: Stop Look Plan			To escape a rip swim parallel to the beach			Give an F about the flags			No flags no swim			Don't put your life on the line		
Awareness	Yes	No	*X* ^2^ (*p*)	Yes	No	*X* ^2^ (*p*)	Yes	No	*X* ^2^ (*p*)	Yes	No	*X* ^2^ (*p*)	Yes	No	*X* ^2^ (*p*)	Yes	No	*X* ^2^ (*p*)
Overall	68.0	32.0	192.89 (*p* < 0.001)	10.2	89.8	945.63 (*p* < 0.001)	36.1	63.9	115.20 (*p* < 0.001)	9.0	91.0	1003.76 (*p* < 0.001)	53.3	46.7	6.56 (*p* = 0.01)	22.7	77.3	444.30 (*p* < 0.001)
Gender																		
Female	74.9	25.1	35.01 (*p* < 0.001)	9.1	90.9	2.37 (*p* = 0.12)	36.1	63.9	0.00 (*p* = 0.987)	7.9	92.1	2.48 (*p* = 0.116)	58.3	41.7	15.90 (*p* < 0.001)	23.1	76.9	0.09 (*p* = 0.764)
Male	60.6	39.4		11.5	88.5		36.1	63.9		10.2	89.8		48	52		22.4	77.6	
Age group																		
18–29 years	54.9	45.1	62.11 (*p* < 0.001)	15.7	84.3	38.19 (*p* < 0.001)	38.1	61.9	14.26 (*p* = 0.003)	17.1	82.9	38.19 (*p* < 0.001)	48.3	51.7	4.87 (*p* = 0.182)	29	71	28.32 (*p* < 0.001)
30–44 years	61.9	38.1		14.7	85.3		29.8	70.2		10.6	89.4		53.9	46.1		28.4	71.6	
45–64 years	72.6	27.4		6.9	93.1		36.5	63.5		5.4	94.6		53.4	46.6		16.5	83.5	
65+ years	82.7	17.3		3.6	96.4		43.3	56.7		4.7	95.3		57.4	42.6		18.4	81.6	
Born in Australia																		
Yes	68.5	31.5	1.15 (*p* = 0.283)	10.9	89.1	3.54 (*p* = 0.060)	37.9	62.1	10.07 (*p* = 0.002)	9.4	90.6	1.05 (*p* = 0.305)	53.8	46.2	0.74 (*p* = 0.390)	23	77	0.24 (*p* = 0.62)
No	65	35		6.9	93.1		27.2	72.8		7.3	92.7		50.8	49.2		21.5	78.5	
Rurality of residence																		
Metropolitan	65.2	34.8	13.98 (*p* < 0.001)	10.5	89.5	0.30 (*p* = 0.585)	34.5	65.5	4.74 (*p* = 0.030)	9.8	90.2	2.68 (*p* = 0.101)	52.8	47.2	0.38 (*p* = 0.535)	22.9	77.1	0.06 (*p* = 0.808)
Regional	75.4	24.6		9.5	905		40.6	59.4		7.0	93.0		54.6	45.4		22.3	77.7	
Summer water usage frequency																		
Frequently (daily or 2–3 times a week)	58.6	41.4	34.23 (*p* < 0.001)	17.4	82.6	54.53 (*p* < 0.001)	38.4	61.6	2.01 (*p* = 0.365)	14.6	85.4	33.81 (*p* < 0.001)	47.9	52.1	14.58 (*p* = 0.001)	29.0	71.0	22.29 (*p* < 0.001)
Infrequently (weekly to monthly)	73.0	27.0		7.6	92.4		34.9	65.1		6.5	93.5		58.2	41.8		20.5	79.5	
Did not use	74.1	25.9		1.0	99.0		34.3	65.7		3.5	96.5		49.8	50.2		14.4	85.6	

Among the coastal‐specific messages, there was significant variation in awareness by age with older age groups (45–64 years and 65+ years) more likely to be aware of ‘Swim Between the Red and Yellow Flags’ (*X* ^2^ = 62.11; *p* < 0.001) and ‘To Escape a Rip, Swim Parallel to the Beach’ (*X* ^2^ = 14.26; *p* = 0.003), while younger age groups (18–29 years and 30–44 years) were more likely to be aware of ‘Give an F About the Flags’ (*X* ^2^ = 38.19; *p* < 0.001) and the ‘The Think Line’ (*X* ^2^ = 38.19; *p* < 0.001) (Table 3).

Awareness of ‘Swim Between the Red and Yellow Flags’ was significantly lower among males (*X* ^2^ = 35.01; *p* < 0.001) when compared to females and higher among regional dwelling respondents (*X* ^2^ = 13.98; *p* < 0.001) when compared to those living in metropolitan areas. Those born outside of Australia (*X* ^2^ = 10.073; *p* = 0.002) and regional resident respondents (*X* ^2^ = 4.74; *p* = 0.030) were more likely to be aware of the message ‘To Escape a Rip, Swim Parallel to the Beach’ (Table 3).

For non‐coastal specific messages, age had a significant impact on awareness for ‘Make the Right Call’ only with higher awareness among the two youngest age groups, 18–29 years and 30–44 years (*X* ^2^ = 28.96; *p* < 0.001). Gender had a significant impact on awareness of ‘Make The Right Call’ (*X* ^2^ = 13.55; *p* < 0.001) and ‘Respect the River’ (*X* ^2^ = 4.27; *p* = 0.039), with males more likely to be aware of these messages when compared to females (Table 4).

**TABLE 4:** Awareness of non‐coastal‐specific water safety messages.

	Stop. Look. Stay Alive			Float to Survive			Make the Right Call			Respect The River			Keep Watch		
Awareness	Yes	No	*X* ^2^ (*p*)	Yes	No	*X* ^2^ (*p*)	Yes	No	*X* ^2^ (*p*)	Yes	No	*X* ^2^ (*pp*)	Yes	No	*X* ^2^ (*p*)
Overall	34.4	65.6	145.88 (*p* < 0.001)	21.8	78.2	475.35 (*p* < 0.001)	13.5	86.5	796.18 (*p* < 0.001)	19.0	81.0	574.80 (*p* < 0.001)	27.4	72.6	304.77 (*p* < 0.001)
Gender															
Female	33.8	66.2	0.23 (*p* = 0.630)	21.5	78.5	0.09 (*p* = 0.769)	10.4	89.6	13.55 (*p* < 0.001)	17	83	4.27 (*p* = 0.039)	29.4	70.6	3.14 (*p* = 0.076)
Male	35	65		22.1	77.9		16.9	83.1		21.2	78.8		25.3	74.7	
Age group															
18–29	38.8	61.2	6.78 (*p* = 0.079)	23.1	76.9	9.34 (*p* = 0.025)	18.9	81.1	28.96 (*p* < 0.001)	17.1	82.9	1.85 (*p* = 0.605)	31.5	68.5	10.39 (*p* = 0.016)
30–44	36.5	63.5		21.6	78.4		18.1	81.9		20.2	79.8		30.7	69.3	
45–64	32.7	67.3		18.1	81.9		8.7	91.3		20	80		25.4	74.6	
65+	29.6	70.4		27.4	72.6		9.4	90.6		17.3	82.7		21.7	78.3	
Born in Australia															
Yes	34.7	65.3	0.28 (*p* = 0.599)	22.7	77.3	3.87 (*p* = 0.049)	13.9	86.1	1.14 (*p* = 0.285)	20.2	79.8	6.86 (0.009)	26.9	73.1	1.04 (*p* = 0.307)
No	32.9	67.1		17.1	82.9		11.4	88.6		13	87		30.1	69.9	
Rurality of residence															
Metro	33	67	3.31 (*p* = 0.068)	21.7	78.3	0.02 (*p* = 0.888)	13.8	86.2	0.25 (*p* = 0.618)	17	83	10.95 (*p* = 0.001)	27	73	0.36 (*p* = 0.594)
Regional	38.1	61.9		22.1	77.9		12.8	87.2		24.6	75.4		28.6	71.4	
Summer water usage frequency															
Frequently (daily or 2–3 times a week)	40.9	59.1	26.61 (*p* < 0.001)	26.1	73.9	9.16 (*p* = 0.010)	20.9	79.1	39.50 (*p* < 0.001)	21.8	78.2	6.97 (*p* = 0.031)	29.0	71.0	9.50 (*p* = 0.009)
Infrequently (weekly to monthly)	33.3	66.7		19.1	80.9		9.4	90.6		18.5	81.5		28.7	71.3	
Did not use	20.9	79.1		20.4	79.6		9.0	91.0		13.4	86.6		18.4	81.6	

Those born in Australia were more likely to be aware of the ‘Respect the River’ (*X* ^2^ = 6.86; *p* = 0.009) and ‘Float to Survive’ (*X* ^2^ = 3.87; *p* = 0.049) messages than those born overseas. Country of birth had no significant impact on awareness of any of the other messages. Those residing in regional areas were more likely to be aware of the ‘Respect the River’ program than those residing in metropolitan areas (*X* ^2^ = 10.95; *p* = 0.001). Awareness of several messages was higher among those who reported frequently using the water over the Summer: ‘Stop. Look. Stay Alive.’ (*X* ^2^ = 6.61; *p* < 0.001), Make the Right Call (*X* ^2^ = 39.50; *p* < 0.001), Float to Survive (*X* ^2^ = 9.16; *p* = 0.010) and Keep Watch (*X* ^2^ = 9.50; *p* = 0.009) (Table 4).

## 4 Discussion

Australia has a long history of drowning prevention public education and awareness raising efforts, including numerous water safety messages and campaigns [8, 20]. Recent research has challenged the efficacy of campaigns and awareness raising to make meaningful reductions in drowning numbers [21, 22]—yet to date (and despite significant effort and investment) few evaluations have been conducted to examine message awareness among the general population [10, 11, 12]. This study aimed to conduct an initial assessment of awareness of selected water safety messages, with a secondary aim of determining if the community of NSW felt the number of water safety messages being promoted was confusing. Findings act as one measure of investment effectiveness but also signal where awareness, as the first step in behaviour change, is and perhaps is not reaching the populations at risk of drowning [14].

Overall, 76% of respondents did not consider the number of promoted water safety messages was confusing, acknowledging a diversity of messages is likely needed to mitigate Australia's rising fatal drowning burden across a range of aquatic locations, demographic groups and activity profiles [2, 23]. However, free text responses highlighted that improvements could be made around explaining concepts rather than assuming knowledge and clearly communicating recommended safety actions. This appeared to be a particularly prevalent finding for the 18–29 years age group, whom it has been suggested may have lower affective intensity towards mass marketing than older age groups [24]. However, beyond this specific age group, findings about the need to translate awareness into action are an important consideration when aiming to encourage safer behaviours [22, 25], which will help the sector in its goal of a 50% reduction in fatal drowning by the year 2030 [8].

Message longevity appears to positively impact awareness, with the longest water safety message among those examined in the study, ‘Swim Between the Red and Yellow Flags’, which began in the 1930s, recording the highest levels of awareness at 68%. Lifeguard supervision is a key drowning prevention strategy, with almost all beach drowning deaths in Australia occurring outside of the flags [26]. However, there remains a significant number of unpatrolled beaches in Australia [27] and more recent messages, through campaigns such as ‘The Think Line’ and ‘Stop. Look. Stay Alive.’, have attempted to encourage safer behaviours in the absence of the flags through more diverse messaging than a singular call to action. With awareness of 10% and 34%, respectively, these newer messages, launched in 2016 and 2024, likely need more time to become prominent among the community. Qualitative insights from survey respondents also support the notion that broader campaigns without a singular call to action may need additional assets and communication strategies to help make the advice behind the message clearer.

While results suggest message longevity contributes to increased awareness, evidence from other domains highlights the need for effective policy and supportive environments to sustain awareness over time [28, 29]. However, message longevity can be problematic in instances where research indicates certain messages are no longer best practice. ‘To Escape a Rip, Swim Parallel to the Beach’ was a prominent beach safety message first promoted in Australia in 2009, originating from early rip current studies dating back to the late 1940s [30]. Originally advising beachgoers caught in rip currents to swim parallel to the beach, research later identified that rip current dynamics were multifaceted and floating could be an additional, potentially better alternative [31]. Although either option worked, floating was found to allow for the conservation of energy [32], while swimming parallel could lead to a person swimming against a current, increasing the risk of exhaustion and drowning [33], and as such, the message is no longer actively directly promoted in Australia; instead, beachgoers are asked to ‘know their options’ [34]. However, our study identified 36% of the NSW population remain aware of the message, with significantly higher awareness among older age groups (45 years and over) compared to younger people (18–44 years), those born overseas and living in regional areas. Dedicated research should explore rip current awareness and knowledge of escape strategies against these groups, traditionally at higher risk of rip current drowning [35], to ensure weak swimmers are aware of the guidance on floating.

Awareness of the ‘Swim Between the Red and Yellow Flags’ message was significantly lower among males when compared to females. This is concerning given males are significantly overrepresented in fatal and non‐fatal drowning statistics in Australia [2, 36] including beach drowning fatalities [26]. It is unclear why such gender differences occur, given the ‘Swim Between the Red and Yellow Flags’ message is widely available to the general public [23]. Future research could be conducted to determine the reasons behind this gender difference, including whether lower risk‐taking around water among women, or potentially greater supervisory roles among women, necessitate a need to find safer places to swim for their children, may be influencing the results of the current study.

Conversely, we identified higher awareness among males when compared to females for the ‘Make the Right Call’ and ‘Respect the River’ messages. ‘Make the Right Call’ in particular focuses specifically on encouraging safer male behaviours around the water and highlights the disparity in drowning risk between males and females [37]. Our findings suggest the benefits in awareness associated with a longstanding message aimed at the general population, as is the case for ‘Swim Between the Red and Yellow Flags’, may not achieve cut through with some harder to reach high‐risk groups, such as males [38].

As an aquatic environment, rivers are a leading location for drowning in Australia, accounting for an average of 73 deaths (including most floodwater‐related drowning deaths) each year over the past decade [2]. ‘Respect the River’ remains the only river‐focused water safety message promoted nationally across Australia, having been in existence since 2015. The current study shows awareness of the ‘Respect the River’ message is higher among males, those born in Australia, and those residing in regional areas, positive findings given all three groups are known to be at increased river drowning risk [39]. However, given significant river drowning burden (22% of all drowning deaths in the 2025/26 financial year [2]), further investment and enhanced understanding of what constitutes effective river drowning prevention approaches should be prioritised.

While awareness is important, there remains a known gap between awareness and actual behaviour [25]. Future research could be conducted to better understand which promotional mechanisms and other more interactive strategies (e.g., [22]) have been successful in achieving higher awareness among at‐risk groups. Beyond awareness, drowning prevention researchers and practitioners should continue to advocate for supportive environments [40], healthy public policy and strategies to increase personal skills, to enhance the efficacy of awareness raising activities in achieving safer behaviours around all waterways.

## 5 Strengths and Limitations

This study represents a first step in assessing awareness of water safety and drowning prevention measures. Such assessment, as recommended by the WHO, can support evaluation of investment and addresses a gap in the research literature. The use of a representative sample from a highly populous jurisdiction is another strength of the study, allowing findings to be extrapolated to the NSW population. There are, however, limitations associated with this research that must be considered. The messages assessed within this study are not an exhaustive list. Respondent gender was collected by dichotomising to binary gender of male/female and therefore may not reflect consideration from LGTBQI+ communities within NSW. Remoteness of respondent's residential location was calculated based on the respondent's postcode and may not represent actual personal living circumstances. The study did not ask participants born overseas how long they had lived in Australia. As length of residence may have influenced the findings, the absence of this data point is a study limitation. Awareness of messages was based on self‐reported data and may be prone to recall bias. We did not examine advertising spend or marketing approaches used to promote messages, and those more recently or heavily promoted over the most recent Summer may be more likely to be recalled among the cohort surveyed. Awareness may not translate to behaviour [21], and the survey did not examine impacts of awareness on self‐reported behaviour. Similarly, awareness may not indicate accurate understanding of intended meaning as was implied in qualitative findings regarding messages that lacked detail or used language that was not easily understood.

## 6 Conclusion

This study adds to the limited evaluation of drowning prevention messages in the Australian setting which can inform future effort, research and resource allocation. While awareness of some messages was higher than others, and higher among groups at known increased risk of drowning for some messages, awareness does not constitute behaviour change. This highlights the importance of a multi‐faceted approach to drowning prevention which includes improving personal skills, supportive environments and health public policy. With rising numbers of drowning fatalities in Australia, ensuring awareness translates into safer behaviours in situ will be vitally important.

## Author Contributions

R.J.I. developed the data collection tool with assistance from A.E.P. Data analysis was conducted by R.J.I. and A.E.P. Manuscript was drafted by A.E.P. with review and editing from R.J.I. and J.C.L. All authors approve the submitted manuscript.

## Funding

This work was supported by Premier's Department, NSW Government (no grant ID). Author A.E.P. is supported by a National Health and Medical Research Council Fellowship (APP ID: APP2009306).

## Ethics Statement

Human research ethics approval was granted by UNSW Sydney Human Research Ethics Committee (approval number: iRECS8327). Informed participant consent was gained prior to the administration of the survey.

## Conflicts of Interest

J.C.L. is employed by Surf Life Saving Australia. A.E.P. holds an honorary (unpaid) appointment with Royal Life Saving Society—Australia.

## Data Availability

The data that support the findings of this study are available on request from the corresponding author. The data are not publicly available due to privacy or ethical restrictions.
